# Structure and Properties of ZrON Coatings Synthesized by Cathodic Arc Evaporation

**DOI:** 10.3390/ma14061483

**Published:** 2021-03-18

**Authors:** Alexander S. Kuprin, Adam Gilewicz, Tatyana A. Kuznetsova, Vasilina A. Lapitskaya, Galina N. Tolmachova, Bogdan Warcholinski, Sergei M. Aizikovich, Evgeniy V. Sadyrin

**Affiliations:** 1 National Science Center Kharkov Institute of Physics and Technology, 61108 Kharkov, Ukraine; kuprin@kipt.kharkov.ua (A.S.K.); tolmachovagn@kipt.kharkov.ua (G.N.T.); 2 Faculty of Mechanical Engineering, Koszalin University of Technology, 75-453 Koszalin, Poland; adam.gilewicz@tu.koszalin.pl (A.G.); bogdan.warcholinski@tu.koszalin.pl (B.W.); 3 Nanoprocesses and Technology Laboratory, A.V. Luikov Heat and Mass Transfer Institute of the National Academy of Sciences of Belarus, 220072 Minsk, Belarus; t_kuzn@hmti.ac.by (T.A.K.); lapitskayava@hmti.ac.by (V.A.L.); 4 Research and Education Center “Materials”, Don State Technical University, 344003 Rostov-on-Don, Russia; s.aizikovich@sci.donstu.ru

**Keywords:** ZrON, coatings, hardness, adhesion, microstructure, roughness, nanoindentation

## Abstract

The transition metal oxynitrides are a coating material with decorative features due to their adjustable color and good mechanical properties. The purpose of the research was to study the effect of the relative oxygen concentration O_2(x)_ = O_2_/(N_2_ + O_2_) in particular on adhesion, but also on the color, structural and mechanical properties of ZrON coatings synthesized by cathodic arc evaporation on HS6-5-2 steel substrates. The surface morphology, phase and chemical composition and mechanical properties were determined using scanning electron microscopy, X-ray diffraction, wavelength dispersive X-ray spectroscopy, nanoindentation and scratch test. It was found that color of the coatings changed from light yellow for ZrN first to gold and then to graphite for Zr-O phase with increase of oxygen concentration. X-ray diffraction patterns showed that the phase separation of ZrN and ZrO_2_ occurred for about 35 at.% of oxygen in the coating. Increase in oxygen concentration in the coatings led to decrease in crystallite size from about 20 nm for ZrN to about 5 nm for coatings with about 35 at.% of oxygen and about 25 at.% of nitrogen. An increase in hardness from about 26 GPa for ZrN to about 30 GPa for coating with small concentration of oxygen (about 9 at.%) and then decrease to about 15 GPa was observed. Adhesion of Zr-O-N coatings demonstrated strong dependence on oxygen concentration. Critical load for ZrN is about 80 N and decreases with oxygen concentration increase to about 30 N for ZrO_2_.

## 1. Introduction

An improvement of the visual characteristics of the surface of different details can be achieved by modifying them with thin decorative coatings. They increase the added value of commercial goods [1]. The most important specifications for this group of coatings are good mechanical and chemical properties, including corrosion resistance and especially attractive and adjustable color. Among the most well-known and recognizable decorative coatings are zirconium nitride and titanium nitride [2], characterized by a golden color. However, the color of the above coatings can only be controlled to a narrow extent. Another group of coatings with variable color are transition metal oxides such as TiO_2_ and ZrO_2_. Their color is caused by the phenomenon of optical interference depending on the thickness of the coatings [1]. The small thickness of coatings significantly limits their usability as decorative coatings. Hence the requirement for a new class of decorative coating materials with adjustable coloration arises.

Adding small amounts of new elements in various forms to protective coatings can significantly improve their mechanical and tribological characteristics [3,4,5]. Expansion of this complex by adding the desired color makes the protective coatings multifunctional. The investigations indicate importance of the effect of the coating’s microstructure on its color. The introduction of new elements to the transition metal nitride is important [2,6,7,8,9,10,11]. Due to their unique properties, mainly optical, of oxygen-doped transition metal nitrides, this new group of functional coating materials has been under investigation for several years. These materials have strength properties between metallic compounds of transition metal nitrides and transition metal oxides [12]. Oxygen has a higher reactivity than nitrogen. The formation of metal-oxygen ionic bonds present in the matrix of the metal-nitrogen covalent bond occurs even for a small oxygenconcentration in the presence of a growing metal nitride layer. The best known compounds are TiO_x_N_y_ and ZrO_x_N_y_, which have different colors associated with oxygen content [6,13,14]. The color ranges from golden yellow with low oxygen to dark blue with high oxygen content [1,13]. Monitoring and selection of nitrogen and oxygen partial pressures enables the production of new structures with different properties [14]. It should be noted that in comparison with transition metal nitrides, much less investigations were conducted on transition metal oxynitrides.

Decorative thin coatings such as ZrON are showing increasing commercial importance. They can be used on eyeglass frames, wristbands and wristwatch casings [13,15]. Such coatings should be scratch and corrosion resistant. ZrON coatings can be manufactured by different PVD processes: Magnetron sputtering [3,13,16,17], ion plating [1,18], arc evaporation [19,20].

The hardness of “pure” ZrN is about 26 GPa [18,21]. Mechanical characteristics of ZrON coatings synthesized using magnetron sputtering are not very high [13]. Hardness is relatively low, from about 7 GPa to about 14 GPa. The values of Young’s modulus range from about 130 GPa to 190 GPa. Both parameters depend on the coating composition. Despite the rather low values observed usage of decorative coatings is possible. A small Young’s modulus may reduce undesirable stresses between the coating and the substrate [13]. Carvalho et al. [22] investigated ZrON coatings formed by rf reactive magnetron sputtering. They found significantly higher values of hardness ranges from 33 GPa for low oxygen fraction to 25 GPa for high oxygen fraction. Additionally, they stated that hardness is almost independent on temperature to about 800 °C. This statement was confirmed by da Silva Oliveira et al. [23] who found an improvement in thermal stability of ZrON coatings with small concentration of oxygen in the ZrN lattice. Due to it the oxidation temperature for H_2_/He atmospheres and vacuum increases to 900 and 800 °C, respectively.

Investigations performed by Vaz et al. [21] indicate that even small addition of oxygen in ZrN results in coating hardness increase. At the same time, oxygen acts as a hardness reducing factor. The increase in oxygen content causes a decrease in the hardness of the ZrON coating. Similar trend was observed for TiON coatings [24]. Huang et al. [1,25] confirmed findings of Vaz et al. [24] about stress decrease as a result of the increase in oxygen flow rate during Zr-O-N coating formation.

The analysis of the literature shows that the oxygen concentration in the coating affects its properties. ZrON coatings are the subject of many publications, but they focus mainly on the optical properties [6,13,19,24,26], structure and morphology of the coatings [1,11,13,17,19,22,24,25,26,27], some mechanical properties (hardness, Young’s modulus, stresses) [1,11,13,18,19,22,27] and electrochemical properties [1]. However, to the best of our knowledge, the adhesion tests of ZrON coatings were not carried out. Since adhesion is one of the main factors determining the functional properties of coatings, it seems advisable to undertake this research topic.

The aim of the study was to evaluate the effect of the relative oxygen concentration O_2(x)_ = O_2_/(N_2_ + O_2_) on the synthesis process, color, the properties of ZrON coatings, both structural and mechanical. The coatings were synthesized by cathodic arc evaporation on HS6-5-2 steel substrates. The coating deposition process was carried at O_2(x)_ = 0%, 10%, 20%, 30%, 50% and 100%.

## 2. Materials and Methods

### 2.1. Coating Deposition

Zr-O-N coatings were produced with various relative oxygen concentration O_2(x)_ = O_2_/(N_2_ + O_2_) by cathodic arc evaporation process using “Bulat-3T” system (Kharkov, Ukraine) [28]. A zirconium cathode (99.9%) with a diameter of 60 mm was used. Substrates of 32 mm in diameter and a height of 3 mm were made from HS6-5-2 steel (ISO 4957) with chemical composition (wt.%), Table 1.

The substrates were ground and polished to a roughness Ra of approximately 0.02 µm. Then they were washed, chemically cleaned in an ultrasonic bath and dried in warm air. The substrates were placed in a holder rotating at 30 rpm at a distance of about 300 mm from the cathode.

After it, the vacuum chamber was pumped to a pressure of 2 mPa. Three processes were carried out in the chamber at the temperature of 350 °C: ion cleaning as an effect of ion bombardment with Zr ions, formation of an adhesive layer and formation of ZrON layer. The technological data are gathered in Table 2.

In determining the samples, the relative oxygen concentration O_2(x)_ during the formation of the coating was taken into account. The coatings were designated ZrO(*x*)N, e.g., ZrO(50)N were synthesized at O_2(x)_ amounted to 50%.

Taking into account the relative oxygen concentration during coating formation, they were marked as ZrO(*x*)N. For example, ZrO(50)N was synthesized at a relative oxygen concentration of 50%.

### 2.2. Coating Characterization

The ball cratering test—standard EN 1071-2: 2002 (Calotest) was applied to evaluate the coating thickness in three randomly selected points.

The chemical composition of the coatings was evaluated using WDS (wavelength dispersive spectrometry) X-ray microanalysis (ThermoScientific’s Magnaray system, thermo Fisher Scientific, Waltham, MA, USA). The accuracy of analyzed elements was ca. 0.5 at.%. The WDS analysis was performed at accelerating voltage 10 kV and the beam current 20 nA. The standards used for analysis were Cr_2_N and Cr_2_O_3_ for nitrogen, and oxygen, respectively. Calculations were performed using the PROZA [29] correction procedure.

To determine the phase composition of the coatings X-ray diffraction (XRD) was applied. The tests were performed on the X’Pert PANalytical diffractometer (Empyrean PANalytical, Malvern Panalytical Ltd., Malvern, UK) using CuKα radiation, a glancing angle (ω = 3°) and Bragg-Brentano geometry in the range of diffraction angles from 20° to 100°. The crystallite size was calculated using the Scherrer formula and Warren-Biscoe correction method [30]. The correction was used due to the incremental diffraction of the diffraction line, approx. 0.2° for the silicon standard. Identification of diffraction lines was performed using the JCPDS database. To assess the preferred orientation of ZrON coatings, the texture coefficient *Tc*(*hkl*) was calculated for each orientation using the equation [31],

(1) $$Tc\left(hkl\right)=\frac{I\left(hkl\right)/I_{o}\left(hkl\right)}{\frac{1}{n}\sum_{1}^{n}I\left(hkl\right)/I_{o}\left(hkl\right)},$$

where:

*I*(*hkl*)—the measured diffraction line intensity,
*I_o_*(*hkl*)—diffraction line standard intensity given in JCPDS database,
*n*—number of diffraction lines analyzed.

The scanning electron microscope JEOL JSM-5500LV (SEM, JEOL Ltd., Tokyo, Japan) and atomic force microscopy (AFM) NT-206 (Microtestmachines, Gomel, Belarus) were applied to investigate the surface microstructure. To determine the statistics of surface defects the metallographic microscope Nikon Eclipse MA200 (Nikon Corporation, Tokyo, Japan) equipped with NIS-Elements (Ar package) was applied. The measurement area was about 430 × 330 μm^2^. The coating surfaces were recorded at the same conditions: magnification (400×) contrast and sharpness. The measurements were performed on five randomly selected areas.

Hardness and Young’s modulus were tested by nanoindentation system Nano Indenter-G200 (Agilent Technologies, Santa Clara, CA, USA) equipped with a three-sided Berkovich intender. The average of ten indentations with standard deviation is presented as a result of measurements. A maximum indentation depth was 300 nm, i.e., less that 1/10 of coating thickness. In the coatings deposited using cathodic arc evaporation a big number of surface defects and related to them high roughness is observed. The requirements concerning correctness of hardness measurement include indentation depth: over 20 × Ra (mean arithmetic roughness) and not more than 1/10 of total coating thickness. In this regard, the reduction of coating roughness was performed to improve the measurement statistics: Coatings were polished with diamond powder to remove the surface defects. This method was previously demonstrated by Romero et al. [32].

The plastic deformation (%) during nanoindentation was used to estimate the plasticity characteristic of ZrON coatings. The plastic deformation (%) is the ratio of total work of indentation to the plastic work. The total and plastic work were measured as the areas under the load curves and between load and unload curves. 24 curves pro sample were obtained via nanoindenter Hysitron 750 Ubi (Bruker, Minneapolis, MN, USA) equipped with the Berkovich indenter with a curvature radius of 100 nm, at the 5 mN load.

The scratch test (Revetest^®^ device, CSM Instruments, Peseux, Switzerland) was applied to assess the coating adhesion. A Rockwell C type diamond indenter with 200 nm radius was used. The stylus moved along the coating surface at a speed of 10 mm/min with a linearly increasing normal load from 0 to 100 N. It was assumed that at the critical load Lc_1_, the first cracks were observed, while for Lc_2_, delamination of the coating was observed. At least three measurements for each coating were carried out. The adhesion was also studied using the Rockwell test [33]. The indented areas were examined using optical microscope and the cracking patterns were observed. It is a test by which the damage to the coating around a indentation is compared with a reference scale. It was assumed that the damage to the coating characteristic for the HF1-HF4 patterns (cracks and fine chips) proves good coating adhesion. Larger coating defects in the tested area, characteristic of the HF5-HF6 patterns, indicate insufficient coating adhesion.

The additional estimation of the crack propagation in Zr-O-N coatings was used via Vickers indentation at the load of 1 N and the subsequent AFM scanning of imprint.

## 3. Results

The coatings tested were characterized by a thickness of approximately 3 μm. A slight reduction in thickness was observed for high relative oxygen concentration during coating deposition. Such a small reduction in the deposition rate with oxygen flow rate rise was observed during formation of ZrON coatings by magnetron sputtering [6,20]. The similar relationship was also observed in the processes of preparation of ZrON coatings by cathodic arc evaporation method [20].

### 3.1. Color of the Coatings

**Figure 1:**
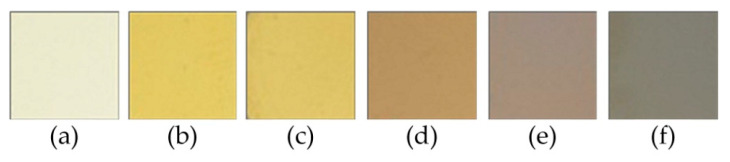
ZrON coatings color for O_2(x)_: (**a**) 0%, (**b**) 10%, (**c**) 20%, (**d**) 30%, (**e**) 50%, (**f**) 100%.

The coatings deposited at a given relative oxygen concentration were characterized by different colors presented in Figure 1. The ZrN coating (Figure 1a) is light yellow. Oxide-doped coatings change color from yellow to graphite, gradually darkening.

The oxygen flow rate increase during the formation of the coatings promotes the substitution of nitrogen by oxygen in the zirconium nitride. This can result in a number of free electrons in the material decrease, and consequently, in the color of the coating modification.

### 3.2. Morphology

**Figure 2:**
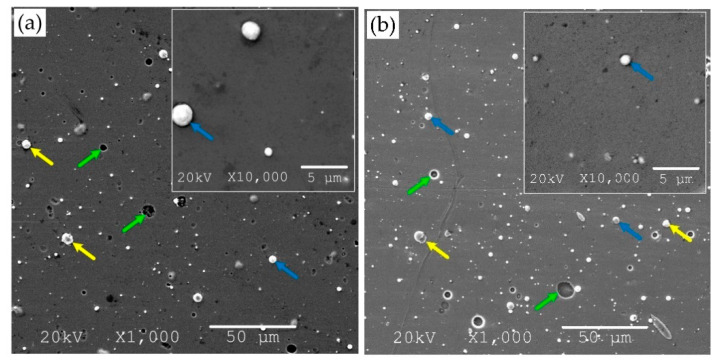

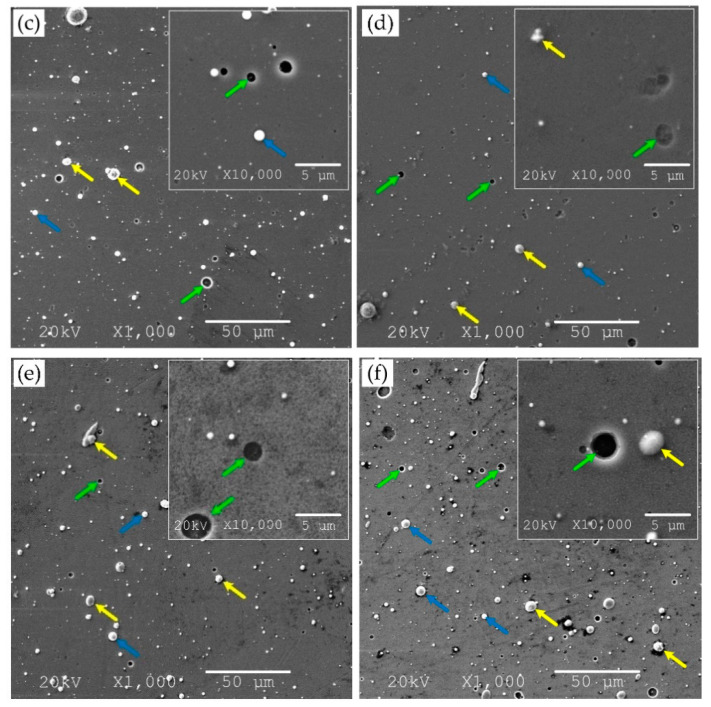
SEM micrograph of Zr-O-N coating morphology, magnification 1000× and 10,000×, the relative oxygen concentration O_2(x)_: (**a**) 0%, (**b**) 10%, (**c**) 20%, (**d**) 30%, (**e**) 50%, (**f**) 100% with marked surface defects—macroparticles (yellow arrows), craters (green arrows), droplets (blue arrows).

As mentioned above, the cathodic arc evaporation method defect is a worse surface quality than in the case of magnetron sputtering [34], manifested by a numerous surface defects. The surface of the ZrON coatings was controlled by the SEM method at magnification of 1000× and 10,000× (Figure 2).

**Figure 3:**
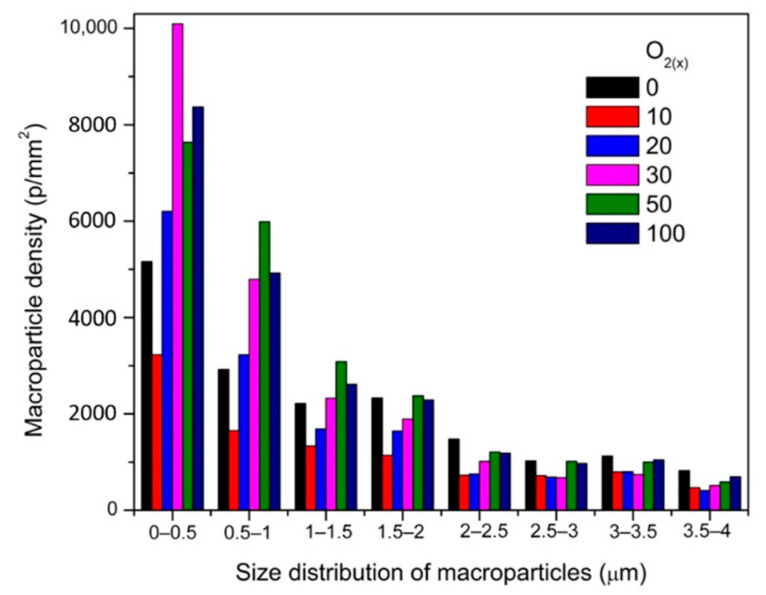
Size distribution and macroparticles density of ZrON coatings synthesized at various O_2(x)_.

An analysis of images of coatings with different oxygen concentration indicated a significant number of surface defects—macroparticles (Figure 2, yellow arrows), craters (Figure 2, green arrows), droplets (Figure 2, blue arrows), etc. Microscopic analysis indicated that the coating obtained at O_2(x)_ = 10% was characterized by the smoothest surface with a low number of fine-sized macroparticles. The vast majority of macroparticles (50–60%) were small, up to 1 μm, Figure 3. 

Particles larger than 3 μm constituted about 7–11%. The amount of macroparticles increased with the relative oxygen concentration increase. A similar effect was previously observed in CrON [34] and ZrON [19] coatings. On the coating surface the craters of various diameters were also apparent (Figure 2, green arrows). Their formation may be related to the removal of poorly bound macroparticles.

**Figure 4:**
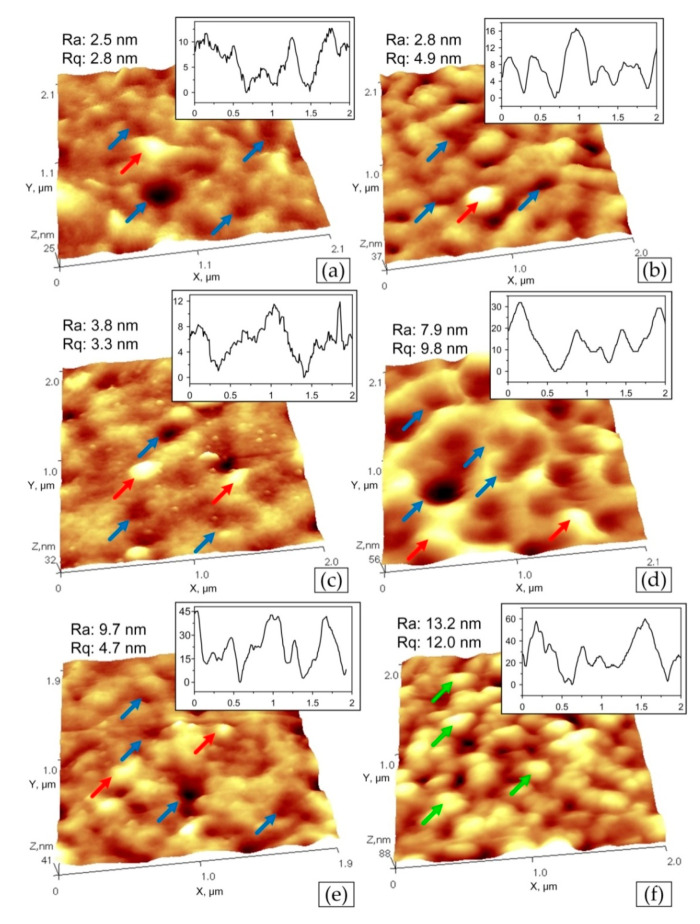
AFM images of Zr-O-N coating morphology with the scan size of 5 × 5 µm^2^, the relative oxygen concentration O_2(x)_: (**a**) 0%, (**b**) 10%, (**c**) 20%, (**d**) 30%, (**e**) 50%, (**f**) 100% with marked surface details—“cells” (blue arrows), submicroparticles (red arrows), grains or microparticles (green arrows).

The flat surface of ZrON coatings investigated by AFM are shown in Figure 4. Roughness parameter Ra of of ZrN coating surface investigated on the area with the size of 2 × 2 µm^2^ is 2.5 nm for ZrN coating, 2.8–9.7 for ZrON coatings and 13.2 nm for ZrO_2_ coating, Figure 4f. Only ZrO(100)N coating has microstructure consisted of grains (or microparticles) with the middle diameter of 200 nm(Figure 4f, green arrows). The other coatings combine “cells” (Figure 4a–e, blue arrows) which are typical for nitride coatings [25] and submicroparticles (Figure 4a–e, red arrows).

**Figure 5:**
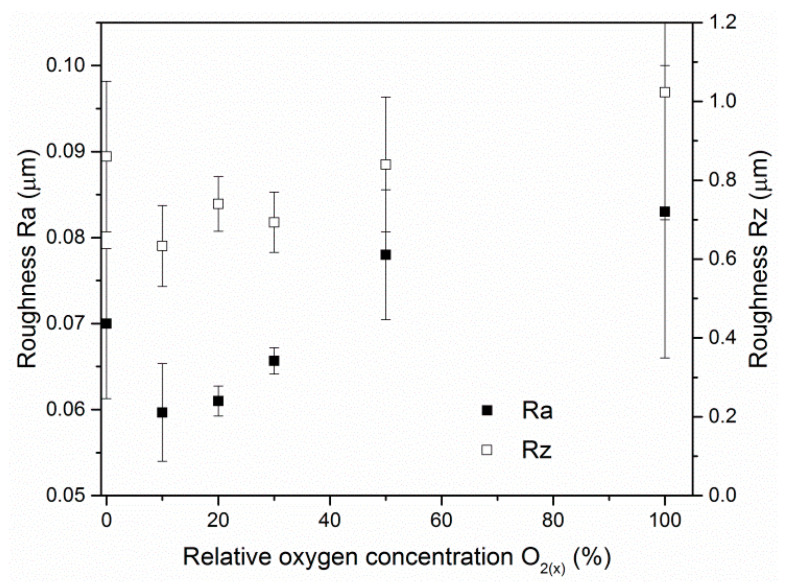
Ra and Rz roughness parameters of ZrON coatings formed at different relative oxygen concentration O_2(x)_.

The surface roughness of the ZrON coatings varied as a result of the different deposition conditions. For higher oxygen flow during the coating formation, more macroparticles were observed on its surface. This resulted in increase in the coating surface roughness (Figure 5). The lowest roughness parameter Ra, about 0.06 μm, was observed for the coating formed at O_2(x)_ = 10%. The ZrN coating had a slightly higher roughness parameter Ra, approximately 0.07 μm. The Rz parameter roughness shows a similar relationship. The results above were confirmed by investigations of Vaz et al. [21]. A similar trend in CrON coatings was described in [28,35].

### 3.3. Chemical Composition

**Figure 6:**
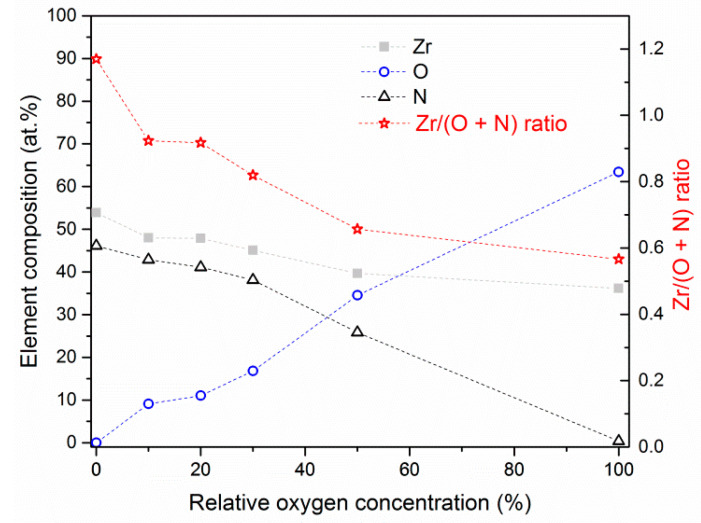
Chemical composition of ZrON coatings formed at various relative oxygen concentrations. Dashed lines are placed only for eyes guiding.

In Figure 6 the chemical composition of ZrON coatings formed at various relative oxygen concentration O_2(x)_ is presented. The following relationship was found: the higher the O_2(x)_ in the working chamber atmosphere during the formation of the coating, the higher the oxygen concentration and the lower zircon and nitrogen concentrations in the coating.

In wide range of O_2(x)_ concentration of all elements changed almost linearly. Only the coating deposited without oxygen, ZrN had a Zr/(O + N) ratio above 1 (1.17), Figure 6. Mentioned ratio decreased to about 0.57 with an increase of relative oxygen concentration during coating formation. This ratio relates to 36 at.% of zirconium and 64 at.% of oxygen, which corresponds to ZrO_2_ phase. It is worth to note that the coatings obtained at a relatively small relative oxygen concentration, up to 30%, had a similar Zr/N ratio ranged from 0.83 to 0.91.

### 3.4. Structure

**Figure 7:**
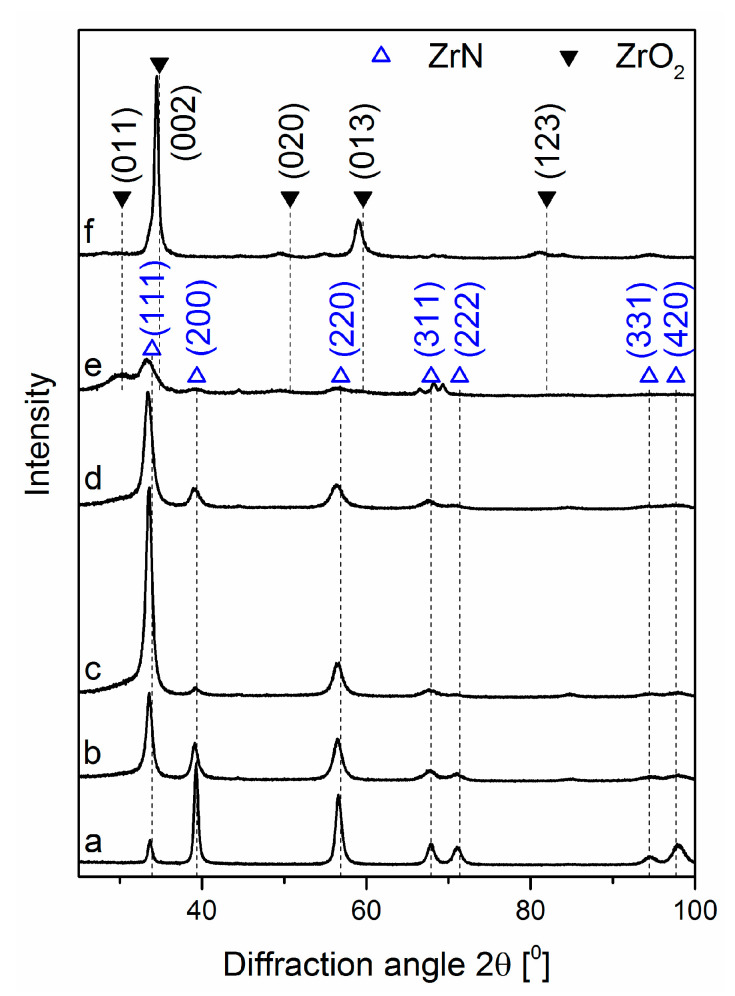
XRD patterns of ZrON coatings synthesized at various relative oxygen concentration O_2(x)_, (**a**) 0%, (**b**) 10%, (**c**) 20%, (**d**) 30%, (**e**) 50%, (**f**) 100%.

The relationship between the texture of the coatings and the relative oxygen concentration was revealed by their structural characterization, presented in XRD diffraction patterns, Figure 7. Diffraction lines characteristic for the cubic ZrN phase were observed, Figure 7a–e. The diffraction line originating from planes (200), Figure 7a, was characterized by the highest intensity.

According to the standard (ICDD 35-0753) the line from planes (111) should be the most intense one. Also, line (220) has a higher intensity for the standard. The intensity of (311), (222), (331), (420) and (111) ZrN diffraction lines are similar, Figure 7a.

The coatings formed with increasing relative oxygen concentration demonstrated differences in diffraction patterns. For O_2(x)_ = 10%, the dominant diffraction line changed. The intensity of the line (111) increased significantly, and the intensity of the line (200) decreased. Diffraction lines for diffraction angle 2θ greater than 60° disappeared. No diffraction lines were observed from oxide phases. Probably, with the increasing amount of oxygen, it may be at the grain boundaries and/or in the amorphous oxide phases. This was predominantly visible in the coating synthesized at O_2(x)_ amounted to 50%. ZrN crystallite size decreased with the oxygen fraction increase. This was clearly observable for the diffraction line (220). The coatings synthesized at a relative oxygen concentration of not less than 50% demonstrated the existence of diffraction lines only from tetragonal zirconia ZrO_2_ (ICDD 50-1089), Figure 7e,f.

The relative oxygen concentration increase during the coatings formation promoted texturing of the coatings, Table 3. The Tc (hkl) value amount to 1 indicates the random packing of crystallites in the coating, while the higher values indicate the preferential orientation of the grains in a given direction (hkl). The results below indicated that the value of the relative oxygen concentration during the coating deposition was critical to the structure and preferred orientation of the crystallites in the coating.

In Zr-N system, many stable compounds such as stoichiometric ZrN and substoichiometric Zr_2_N, Zr_4_N_3_, Zr_6_N_5,_ Zr_8_N_7_ are present. The lowest enthalpy of formation shows the rocksalt ZrN with space group $Fm\bar{3}m$ [36]. Presented here diffraction patterns (Figure 7) did not reveal the presence of above compounds except ZrN.

**Figure 8:**
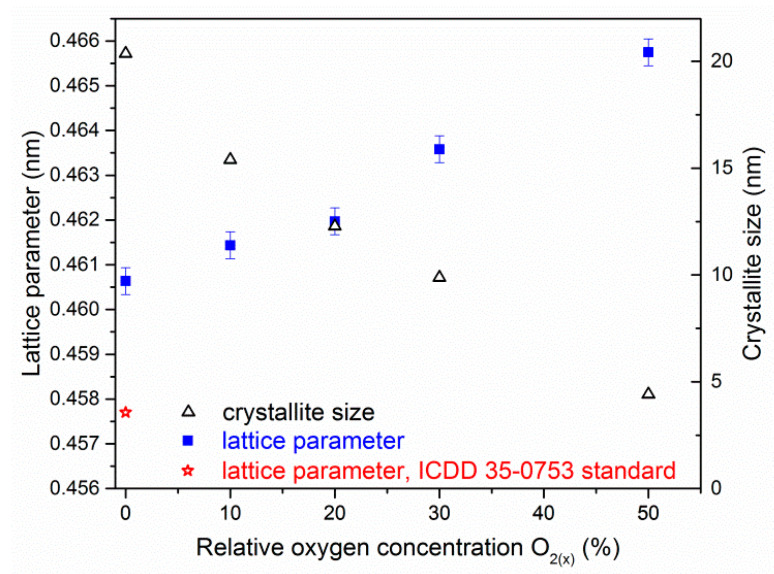
Lattice parameter and crystallite size of ZrON coatings dependent on O_2(x)_.

The reduction of the intensity and broadening the diffraction lines as well as the shift towards lower angles with relative oxygen concentration increase was observed. This suggests that the crystallite size decreased, as well as the stress in the coatings due to oxygen binding to the interstitial positions increased. This may lead to gradual amorphisation of the coating, Figure 7e. The change in chemical composition of ZrON coatings results in a change in the lattice constant, which increases almost linearly with relative oxygen concentration, Figure 8.

The ZrN lattice parameter is 0.4577 nm according to ICDD 35-0753. The lattice constant of the tested ZrN coating was larger than the standard and it was (0.4606 ± 0.0003) nm. This might be due to stresses in the coating resulting from ion bombardment of the surface of the coating during deposition, as well as the elevated temperature of forming the coating.

For ZrN coating the size of crystallite is about 20 nm and decreases to about 5 nm for ZrO(50)N coating containing about 35 at.% of oxygen and 25 at.% of nitrogen, Figure 8. Rawal et al. [26] observed that an increase of oxygen concentration in ZrON magnetron sputtered coatings leads to decrease of crystallite size, to 10–12 nm.

### 3.5. Hardness

**Figure 9:**
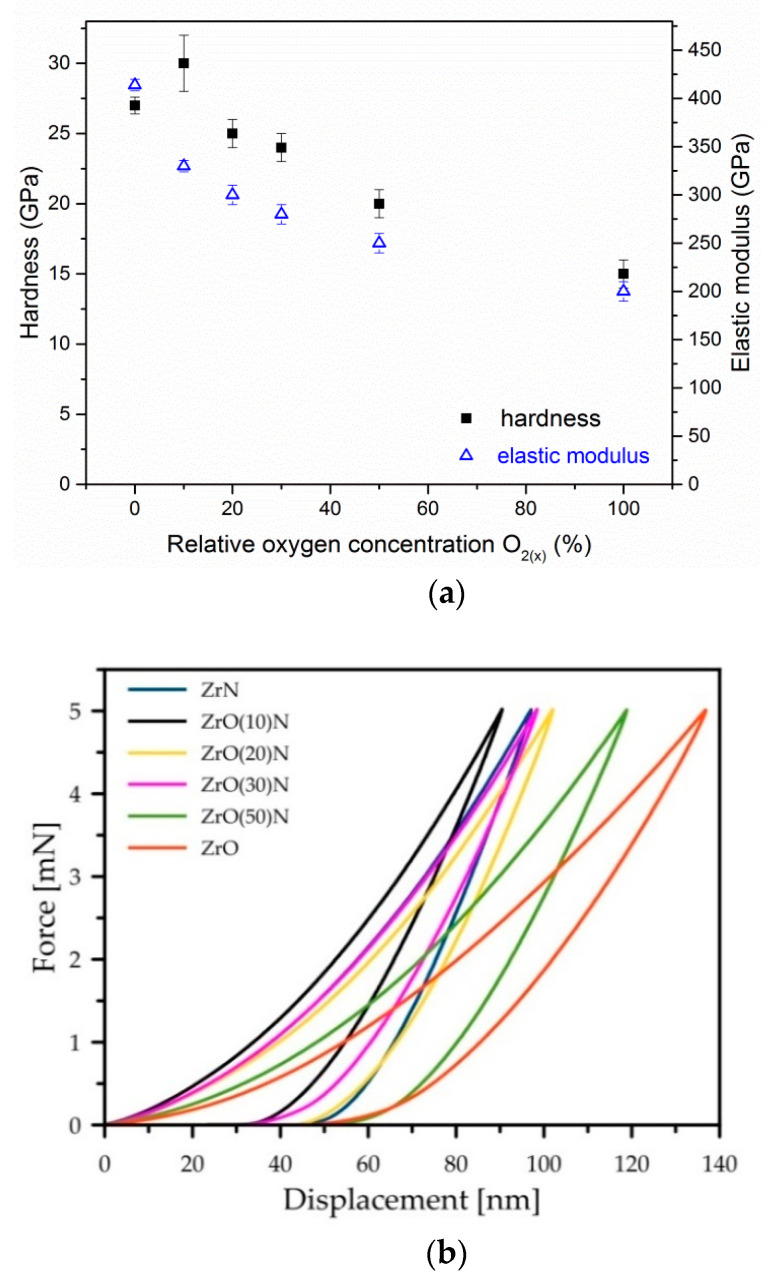

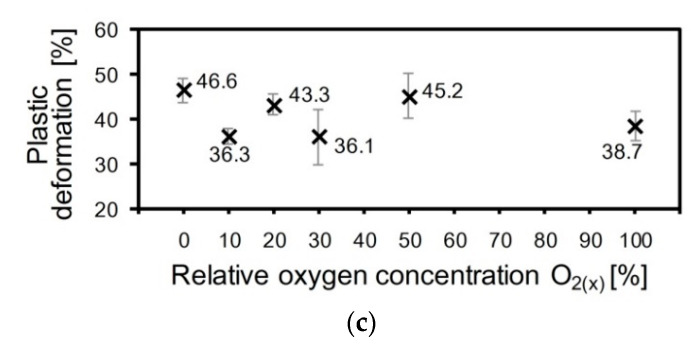
The dependences of hardness and Young modulus (**a**), indentation curves shape (**b**) and plastic deformation (**c**) of ZrON coatings with different O_2(x)_.

The hardness and elastic modulus evolution related to relative oxygen concentration during coating deposition is presented in Figure 9a. The hardness of ZrON coatings decreases almost linearly with the increase in O_2(x)_. The hardness of ZrN and ZrO_2_ phases was about 27, and 15 GPa, respectively. The coating deposited at O_2(x)_ = 10% is characterized by the largest hardness, about 30 GPa. This is probably related to the solid solution hardening mechanism. Lattice deformation due to oxygen doping may result in higher material strength.

According to the shape of the nanoindentation curves, the plastic deformation of the Zr-O-N coatings does not depend on the oxygen content (Figure 9b,c). The “high” values (43.3–46.6%) were determined for ZrN, ZrO(20)N and ZrO(50)N. The “low” values (36.1–38.7%) were measured for ZrO_2_, ZrO(10)N and ZrO(30)N.

### 3.6. Adhesion

**Figure 10:**
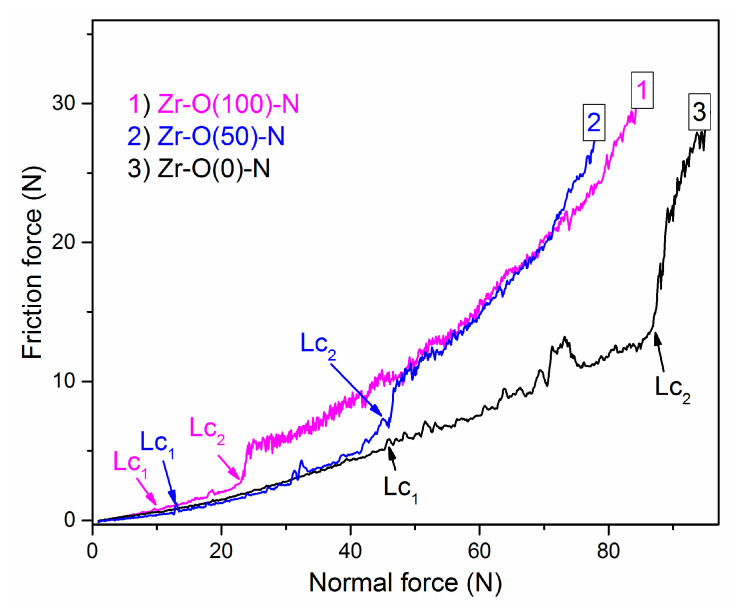
The friction force in scratch test on ZrON coatings with different O_2(x)_ plotted versus the applied load.

Adhesion is a key feature of the coating from the point of view of its usability. One of the commonly used, effective and fast methods of achieving critical loads connected with the coating adhesive properties is the scratch test. The courses of friction force versus normal force for selected coatings are presented in Figure 10. ZrN coating was characterized by the highest adhesion, on the other hand, ZrO_2_ zirconium oxide showed the lowest adhesion. The zirconium oxynitride had an intermediate value.

The critical force depends on various factors: Loading rate, stylus-tip radius, roughness, hardness of the substrate and the coating, coating thickness, friction between stylus-tip and coating [37,38]. Due to this, all scratch tests were performed in similar conditions on the coatings characterized by similar thickness of about 3 µm.

**Figure 11:**
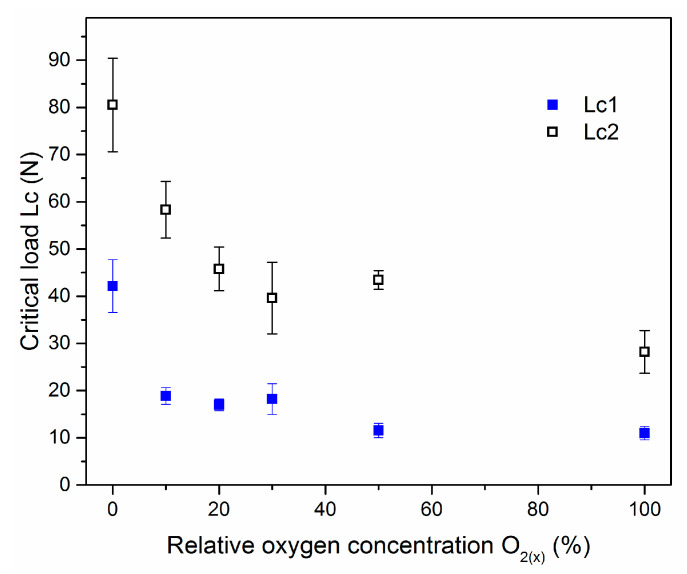
The critical loads for Zr-O-N coatings deposited at different O_2(x)_.

Critical load values, Lc_2_ and Lc_1_, are presented in Figure 11. One can observe the both critical loads decrease with relative oxygen concentration increase (Figure 10). Due to lower critical load of transition metal oxides on steels compared to metal nitrides observed relation seems to be obvious. Lower critical load for ZrO_2_ coatings compared to ZrON ones was presented previously in [38,39]. CrON coatings synthesized by a conventional arc evaporation were studied by Gautier and Machet [40]. They observed similar trend in mechanical properties of above coatings. They observed a decrease of critical load with O_2(x)_. Other elemental composition of the coatings or modification of their structure may be the reason for this [40].

**Figure 12:**
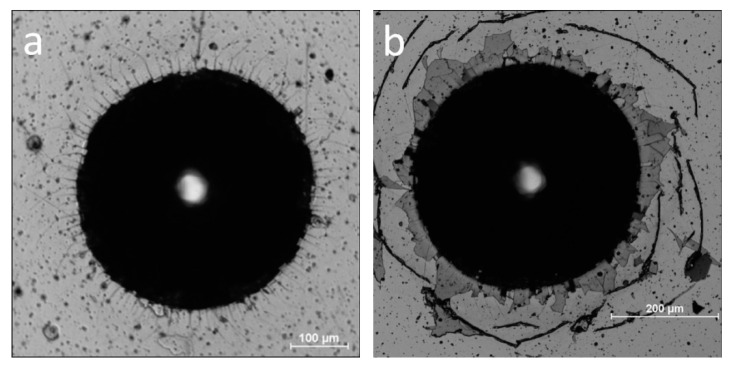

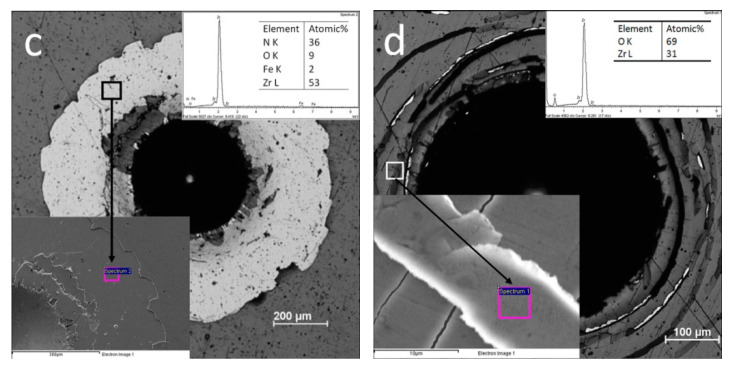
Micrograph of the indentation morphology in Rockwell test: (**a**) ZrO(0)N, (**b**) ZrO(10)N, (**c**) ZrO(20)N, and (**d**) ZrO(100)N.

To study the failure mechanism and coating adhesion strength VDI Guideline 3198 [33] procedure was used. It is an easy-to-apply and fast adhesion test for hard coatings synthesized on hardened steel substrates. The micrographs of the indentations are presented in Figure 12. The presence of little or no fracture radial lines denote good cohesion and no delamination on good adhesion of ZrO(0)N coating, Figure 12a.

For ZrO(10)N coating, cracks appear around the indentation, Figure 12b. They are connected with the pile up of substrate material as a result of indentation and indicate on big tensile stresses which may be present near indent. Brittle and hard coatings are characterized by this type of damage [41]. The small amount of short radial cracks point at improved cohesion of the coatings obtained.

ZrO(20)N coating and other samples with higher concentration of oxygen show higher area of delamination, but Zr interlayer still adheres to the substrate. The chemical composition of the area near indentation (see Figure 12c black square and insert in left down corner) is similar to composition of the coating, Zr—48 at.%, O—9 at.%, N—43 at.% (Figure 6). A little higher concentration of zirconium and lower of nitrogen, and presence of small concentration of iron indicate rather on cohesive fracture than adhesive.

In ZrO(100)N coating a distinct circular cracks are visible. The delamination of the coating is not presented. The chemical composition of the marked fragment of the crack (Figure 12d) detected by EDX show zirconium and oxygen in the ratio of about 1 to 2 which corresponds to the stoichiometry of ZrO_2_. Simultaneously, the iron spectrum was not recorded, which indicates the absence the adhesive and cohesive of damages of the coating.

**Figure 13:**
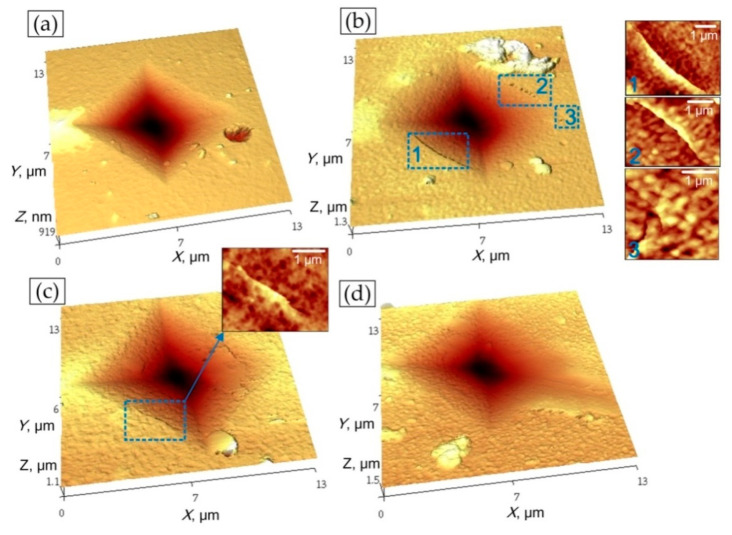
Pictures of failure after Vickers indentations: (**a**) Zr-O(0)-N, (**b**) Zr-O(10)-N, (**c**) Zr-O(50)-N, and (**d**) Zr-O(100)-N.

According the results of Vickers indentation and AFM scans of imprints in ZrN, ZrO_2_, ZrO(10)N and ZrO(50)N coatings cracks around the imprints were discovered only in ZrO(10)N and ZrO(50)N (Figure 13). Wherein in ZrO(10)N four cracks on the opposite sides of imprint were found and in ZrO(50)N only one crack was observed. The lower cracking ability of ZrO(50)N can be explained by its comparatively higher plasticity, defined as plastic deformation (Figure 13). Higher plasticity helps to dissipate the indentation energy and up to a certain load level to prevent the cracks formation.

## 4. Discussion

Cathodic arc evaporation was used to deposit ZrON thin coatings. The pressure of the reactive gas mixture (oxygen and nitrogen) was constant during the deposition. It is mentioned above that a slight decrease in coating thickness was observed with increasing relative oxygen concentration. This is probably related to the poisoning of the target. The melting point of Zr (2128K) is much lower than that of ZrN (3233K) and ZrO_2_ (2950K). Therefore, the target surface can be covered with the oxide compound and zirconium nitride material. Due to the lower sputtering efficiency of zirconium oxide and nitride, the deposition rate was reduced.

The increase in concentration of oxygen in the coating (Figure 6) changes: its structure (Figure 7), the size of the crystallites and the lattice constant (Figure 8) and texture (Table 3). A higher value of the lattice constant is calculated, the strain (ε), $\varepsilon =\;\frac{\Delta d}{d}=\;\frac{d_{o}-\;d_{s}}{d_{s}}$ [27], where *d_o_* is lattice constant observed and *d_s_* is lattice constant standard, ranges from about 0.6% for coatings synthesized at O_2(x)_ = 0% to 1.8% (O_2 (50)_). This may be due to stresses generated in the coating as a result of ion bombardment on its surface. Also, the substitution of nitrogen by oxygen or incorporation of oxygen at interstitial positions can change the lattice constant. The radius of the oxygen atom is about 60 pm, which is smaller than the radius of the nitrogen atom (65 pm). Therefore, the increase in lattice constant suggests rather an interstitial position of oxygen.

Many studies have shown that the diffraction lines shift speak for the occurrence of residual stresses [30]. Hooke’s Law (σ = E ∙ ε) allows the stresses σ to be calculated. They range from about 2.7 GPa for an oxygen free deposited coating to about 4.5 GPa for a coating formed at O_2(x)_ = 50%.

The intensity ratio of diffraction lines (200) and (111) decreases from about 4.5 for the ZrN coating (O_2(x)_ = 0%) to about 0.1 (O_2(x)_ = 50%). In the B1(fcc) CrN structure, the plane (111) is more densely packed than the plane (200). It can be assumed that the surface of the coating with the dominant diffraction line (111) should be more smooth. Farkas et al. [42] found that for magnetron sputtered Zr-N coatings deposited with increasing nitrogen pressure the I_(200)_/I_(111)_ ratio decreases. It improves the smoothness of the coatings. The results gathered in Figure 6 did not confirm Farkas’s observation. This may be connected with different coating deposition method, cathodic arc evaporation, and the occurrence of a great amount of surface defects, deteriorating its quality. The results shown in the Table 3 indicate that the coating was heavily textured with the dominant (111) diffraction line. This may also be the reason for a significant reduction in I_(200)_/I_(111)_ ratio. The presence of oxygen could also increase the surface roughness. Previous authors’ investigations [43] showed that ZrON coatings formed using magnetron sputtering method were characterized by an increasing value of the roughness parameter Ra from 0.088 μm for ZrN, 0.129 μm (ZrON) to 0.145 μm for ZrO_2_.

Difficulties in the formation and movement of dislocations in the crystal lattice, for example stopping their movement at the grain boundaries may increase hardness of material [21]. The hardness investigated is significantly higher than presented by da Silva Oliveira et al. [13]. The similar trend was observed by Vaz et al. for TiON [21] and ZrON [18]. Vaz et al. [21] found that the compressive stresses in the ZrON coatings decrease from about 8 GPa for low oxygen fraction to less than 1 GPa for higher oxygen fraction. The compatibility of hardness and stress for their coatings formed using rf reactive magnetron sputtering is clearly visible. ZrN coating doped by oxygen may induce defects in the structure of the coating. This effect may decrease the dislocation motion, and thus increase the hardness [21]. The coatings synthesized at a higher relative oxygen concentration were characterized by a smaller crystallite size, Figure 8. This can result in two possible effects: formation of amorphous oxide phases and/or amorphization of ZrN phase, Figure 6e. Both of them could explain the hardness reduction.

Discrepancies in the adhesion and hardness of zirconium oxynitride coatings may result from differences in chemical composition and stoichiometry of the coatings. It can be concluded that differences in the chemical composition of ZrON coatings may lead to differences in their mechanical behavior.

Coating crack initiation resistance, denoted as Lc_1_, is an important feature of the coatings. Thin coatings ought to present good adhesion with a high Lc_1_ value. Investigations conducted by Zhang et al. [44] showed that for high coating toughness high Lc_1_ and Lc_2_ (coating delamination) values are necessary. They introduced a new, quick qualitative toughness index for the coatings called Scratch Crack Propagation resistance, CPRs = Lc_1_ × (Lc_2_ − Lc_1_). Using this equation, the highest toughness was calculated for the ZrN coating (1618 N^2^). The above index decreases with oxygen concentration increase and for ZrO(100)N coating reaches the value of 189 N^2^. Indeed, this coating showed the worst adhesion, defined in the Daimler Benz scale as HF6. Gautier and Machet [40] observed a similar tendency to reduce the critical load of CrON coatings formed with an O_2(x)_ increase. As mentioned above, this may be associated with a different chemical composition of the coating and/or modification of the crystal structure. Oxide coatings are characterized by worse adhesion.

The stresses calculated above, from the shift of the diffraction lines, can cause a reduction in coating adhesion and even delamination, as shown in Figure 12.

## 5. Conclusions

Zr-O-N coatings were systematically investigated. They were synthesized by cathodic arc evaporation at different O_2(x)_. The detailed analysis revealed the follow statements:

– the coatings showed good decorative properties. The ZrN coating color is light yellow and darkens with increase of oxygen concentration to graphite for Zr-O phase,
– deposition rate—a decrease in this parameter was observed with an increase in O_2(x)_ during deposition. It is probably connected with the target poisoning,
– the lowest macroparticle density on the surface was for ZrON coatings synthesized at O_2(x)_ = 10%. The surface macroparticle density increased with O_2(x)_,
– in ZrON coatings the texture dependence on the O_2(x)_ was observed. In the coatings formed at low O_2(x),_ ranged from 10% to 30%, no diffraction lines of the Zr-O phases were registered, only the crystal structure from the ZrN phase,
– a small increase in hardness for the coating deposited with the lowest relative oxygen concentration was observed. It can be related to lattice distortions as an effect of oxygen introduction into the ZrN lattice. Then the hardness and elastic modulus decrease with O_2(x)_ increase,
– adhesion of ZrON coatings strongly depended on oxygen concentration. For ZrN coating critical force was about 80 N and decreased to about 26 N for coating deposited at O_2(x)_ = 100%.

## Figures and Tables

**Table 1:** The chemical composition of HS6-5-2 steel.

C	Mn	Si	P	S	Cr	W	Mo	V	Cu	Fe
0.82–0.92	<0.40	<0.50	<0.03	<0.03	3.5–4.5	6–7	4.5–5.5	1.7–2.1	<0.30	balanced

**Table 2:** Parameters for ZrON coatings deposition.

Parameter	Ion Etching	Adhesive Layer	ZrON Coating
Zr cathode current [A]	75	75	75
Argon pressure [Pa]	0.5	0.5	-
Relative oxygen concentration O_2(x)_ = O_2_/(O_2_+N_2_) [%]	-	-	0, 10, 20, 30, 50, 100
Total pressure [Pa]	0.5	0.5	2
Substrate bias voltage [V]	−1300	−100	−100
Deposition time [s]	180	300	1800
Thickness [µm]	-	~0.1	~3

**Table 3:** Texture coefficient Tc(hkl) for the coatings tested.

Coating	(hkl) Plane						
	(111)	(200)	(220)	(311)	(222)	(331)	(420)
ZrO(0)N	0.14	0.83	1.20	0.52	1.23	0.97	2.10
ZrO(10)N	1.06	0.67	1.46	0.52	0.98	1.03	1.28
ZrO(20)N	3.02	0.12	1.30	0.36	0.23	0.87	1.10
ZrO(30)N	1.96	0.42	1.08	0.53	0.61	1.14	1.27
ZrO(50)N	2.23	0.46	0.31	-	-	-	-

## Data Availability

All data were provided in the article.
